# Protective Cultures in Meat Products and Plant-Based Meat Alternatives: Prospects and Challenges

**DOI:** 10.3390/foods15213984

**Published:** 2026-11-06

**Authors:** Tobias Hennes, Martin Wagner, Beatrix Stessl

**Affiliations:** 1 Austrian Competence Center for Feed and Food Quality, Safety and Innovation, Technopark 1D, 3430 Tulln, Austria; tobias.hennes@vetmeduni.ac.at (T.H.); martin.wagner@vetmeduni.ac.at (M.W.); 2 Unit of Food Microbiology, Centre for Food Science and Veterinary Public Health, Clinical Department for Farm Animals and Food System Science, University of Veterinary Medicine, 1210 Vienna, Austria

**Keywords:** protective cultures, plant-based meat alternatives (PBMAs), cooked meat products, food safety

## Abstract

In view of the considerable levels of food loss on a global scale, the attendant greenhouse gas emissions, and the expanding demand for foods that are as natural as possible, bioconservation strategies—protective cultures in particular—are becoming increasingly important in food production. The objective of this review was to present the current state of research on the use of protective cultures and bacteriocins in plant-based meat alternatives as well as in cooked meat products. To this end, a comprehensive review and relevance-based screening of more than 133 records identified through scientific databases was conducted and complemented by 86 legal documents, market analyses, manufacturer information, outbreak reports, and studies addressing probiotics and antimicrobial resistance. Furthermore, market analyses, manufacturer information, legal frameworks, and regulatory information on outbreak incidents were considered. The evaluation incorporated technological aspects and regulatory requirements in order to provide a comprehensive picture of current developments and challenges in this field. A review of the extant literature reveals an absence of studies that examine the utilization of protective cultures in plant-based meat alternatives. However, extant research on microbial diversity suggests that potential protective cultures could include strains similar to those found in conventional meat products, but this still needs to be experimentally validated. Moreover, there is a significant need for research regarding the combination of different protective cultures, particularly in conjunction with bacteriocins. Technologically, application has hitherto been carried out predominantly after heat treatment, although injection prior to heat treatment would be technologically advantageous. In this context, encapsulation is a promising approach that has yet to be thoroughly investigated. A considerable body of research has hitherto been dedicated to the inhibition of *Listeria monocytogenes*. In future, when selecting *Listeria* strains, those associated with foodborne outbreaks should be favored over pure laboratory strains and unspecified wild strains.

## 1. Introduction

The significant negative impact of food waste, particularly in terms of its contribution to the global carbon footprint and climate change, highlights its relevance to the Sustainable Development Goals (SDGs). Not only is addressing this issue a global priority, but it is also the responsibility of the food industry to adopt sustainable practices (United Nations, https://sdgs.un.org/topics/chemicals-and-waste, accessed on 5 November 2026) [1,2].

SDG Target 12 explicitly calls for ‘sustainable consumption and production patterns’ and commits Member States to halving per capita food waste at retail and consumer levels and reducing food losses across production and supply chains by 2030, including post-harvest stages (United Nations, https://sdgs.un.org/topics/chemicals-and-waste, accessed on 5 November 2026) [2].

Consumers and retailers value information about ingredients and additives and are interested in food production. The trend toward products with clean labeling and particularly fresh, minimally processed products is also evident in discount stores and retail chains’ private label programs, as foods that contain many additives must be declared with E numbers (e.g., sorbic acid–potassium sorbate E 200–202) (European Commission, https://ec.europa.eu/food/food-feed-portal/screen/food-additives/search, accessed on 5 November 2026) [3,4].

At the same time, there is a strong demand for products with a long shelf life. Manufacturers should allocate more resources to this demand by monitoring the production of their products (Hazard Analysis and Critical Control Points—HACCP; Good Hygienic Practices—GHPs) and improving their risk analysis of products placed on the market until the end of shelf life and beyond. This enables particularly sustainable but also cost-saving production [5,6].

The rapid global movement of goods, a defining feature of globalization, has further emphasized the need for stable products. This has driven the demand for technological advancements to combat microbial spoilage and contamination by food-pathogenic microorganisms. Starter and protective cultures play a critical role in enhancing food stability and shelf life, acting as biological preservatives by outcompeting spoilage microorganisms and pathogens. It is evident that they constitute a pivotal element within the framework of hurdle technology, thereby serving as a substitute for chemical preservatives [7,8].

The fundamental difference between starter and protective cultures lies in their different technological applications.

Starter cultures are microorganisms intentionally introduced into food to initiate and control fermentation processes. Their application enhances not only the sensory properties of food but also its nutritional value and digestibility [9]. Starter cultures are often used in the production of fermented meat products, including salami, air-dried sausages, and onion sausages. The main function of these enzymes is to accelerate processes, such as pH lowering, thereby influencing the color, texture, and taste of the products and developing their characteristic aroma. In addition, starter cultures have been shown to improve stability, shelf life, and safety [10].

In contrast, protective cultures are microorganisms that suppress the growth of spoilage organisms (e.g., *Brochothrix* (*B*)*. thermosphacta*) and foodborne pathogens (e.g., *Salmonella* and *Listeria* (*L.*) *monocytogenes*) through the production of antimicrobial compounds [11,12].

The use of protective cultures is a practice applied with the intention of extending shelf life, increasing safety, and reducing spoilage. This phenomenon occurs through three different mechanisms: the synthesis of organic acids that lower the pH value, the production of bacteriocins, and competing directly with spoilage organisms for nutrients, which can lead to displacement [13,14].

Bacteriocins are a heterogeneous group of antimicrobial peptides produced by various protective culture strains [15]. The focus of scientific research has predominantly been on bacteriocins produced by LAB, *Bacillus*, or *Staphylococcus* (*S.*) *pasteuri*. Bacteriocins demonstrate efficacy even at low concentrations, exhibit high heat stability, maintain stability at low pH values, and do not affect the nutritional properties of food [16,17,18,19].

In addition to extending shelf life to reduce food waste, lowering meat consumption has emerged as a critical strategy for cutting CO_2_ emissions. Animal product production is particularly carbon-intensive, driving increased efforts to develop plant-based meat alternatives (PBMAs). The growing market for these products is especially notable, with plant-based alternatives projected to capture 25% of the market by 2040 (Statista, https://de.statista.com/outlook/cmo/lebensmittel/fleisch/weltweit#umsatz, accessed on 5 November 2026) [20,21].

Given the increasing market relevance of PBMAs, the potential application of protective cultures in this area is a topic of interest.

In 2025, the global market for protective cultures in the food and beverage sector recorded a revenue volume of $1.5 billion, and this sector is expected to exhibit a compound annual growth rate (CAGR) of between 4.3% and 23.6% by 2033 and 2035, respectively. The most significant economic market for functional foods is currently Europe (Mordor Intelligence™–Industry Reports Source, https://www.mordorintelligence.com/industry-reports/starter-cultures-market, accessed on 5 November 2026; Market Data Forecast, https://www.marketdataforecast.com/market-reports/protective-cultures-market, accessed on 5 November 2026) [22,23].

Despite extensive research into the use of protective and starter cultures in meat and processed cooked meat products, the knowledge and application of protective cultures in PBMAs remain limited (Chr. Hansen, https://mtc.si/food-cultures-and-enzymes-from-chr-hansen/, accessed on 5 November 2026, M-Food, https://www.meatcracks.de/media/m-cultures_meat_de_ex.pdf, accessed on 5 November 2026) [24,25]. Consequently, the use of raw materials and additives, production technologies, and the market landscape in this sector are highly dynamic. It is especially important to consider the matrix, microbiome, and manufacturing processes when using protective cultures in plant-based meat alternatives [26]. To date, no published studies have investigated the use or effectiveness of protective cultures in PBMAs.

This review focuses on the possible parallel and transfer possibilities of existing protective cultures and their bacteriocin derivates from meat products to their plant-based alternatives and considers gaps in the current application of protective cultures in cooked meat products. Valuable insights may be gained in doing so, and key areas that require further investigation can be established.

## 2. Literature Review—Workflow

The main aim of this review is to clarify the current scientific and economic status of protective cultures and bacteriocins for cooked meat products and their plant-based alternatives. In particular, the influence of protective cultures and bacteriocins on product safety, quality, and longer shelf life was examined. Further research has also addressed microbial diversity, the safety of protective cultures, and technological information on the production of meat-based products and plant-based alternatives (Supplementary Figure S1).

For primary web-based research, Scopus, PubMed, and Web of Science were prioritized, to access reviews or bibliometric analyses. Google Scholar research was applied to achieve broader coverage that includes gray literature, preprints, and conference papers. As secondary sources, ScienceDirect, SpringerLink, and Wiley Online Library provide full-text access to a wide range of articles and book chapters. Legal texts and guidelines and outbreaks (*n* = 26) from the relevant authorities are also reflected. The websites and guidelines of manufacturers of protective cultures and trade fair reports (*n* = 6) and market analyses (*n* = 6) were ultimately considered in the review process.

When developing search strings, we applied Boolean operators AND and OR to refine the search and capture all relevant variations in keywords. After an initial search, the number of articles was 1320. After removing duplicates, 1108 studies remained. The aim of the studies and the conclusions of the literature references were examined, and studies were excluded if they were not related to starter cultures, protective cultures, meat, or plant-based products (*n* = 875). After this step, 233 studies remained. Then, studies on raw fermented products and/or starter cultures were examined in more detail with regard to their thematic relevance for this review. Subsequently, studies on starter cultures and fermented products were excluded if the cultures examined in those studies did not show any initial evidence of a protective effect (remaining studies: *n* = 133).

As the study design on protective cultures and bacteriocins was very heterogeneous, a meta-analysis was not possible because the results were not statistically comparable.

We also compiled an overview of the protective cultures and plant-based meat alternatives available on the market (Section 6).

## 3. Protective Cultures

**Figure 1:**
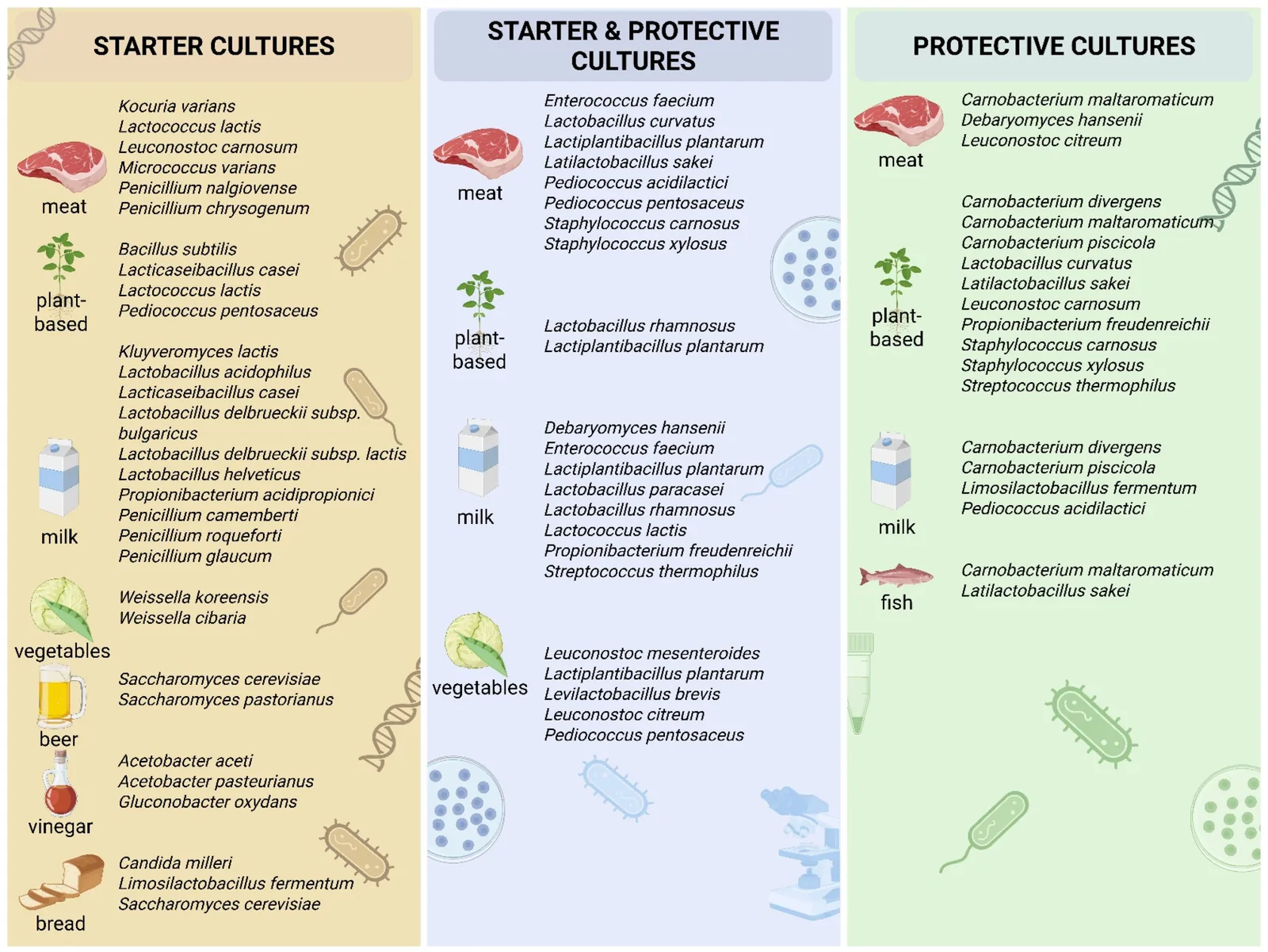
Categorized overview of starter and protective cultures currently applied across different food product categories (U.S. Food & Drug Administration, https://www.fda.gov/files/food/published/GRAS-Notice-GRN-760-Lactobacillus-curvatus-DSM-18775%20.pdf, accessed on 5 November 2026, EFSA, https://www.efsa.europa.eu/en/topics/topic/qualified-presumption-safety-qps, accessed on 5 November 2026) [8,9,10,11,12,27,28,29]. Created in BioRender. Hennes, T. (2026) https://BioRender.com/9y25s3m, accessed on 5 November 2026.

Protective cultures are used as single or mixed cultures with defined physiological and technological properties (Figure 1).

Lactic acid bacteria (LAB), including the genera *Lactococcus*, *Lactobacillus*, *Streptococcus*, *Pediococcus*, *Leuconostoc*, *Enterococcus* and *Weissella*, have played a central role in food fermentation for centuries. Historically, their fermentative activity was used to initiate and drive fermentation processes, and the subsequent development of defined starter cultures enabled them to be used more specifically in controlled food fermentation. In recent decades, the beneficial antimicrobial and protective properties of selected LAB have been recognized, leading to their use not only as starter cultures, but also as protective cultures that inhibit undesirable microorganisms and contribute to the preservation and microbiological stability of foods.

The categories “starter culture” and “protective culture” are not taxonomic in nature, but rather functional and application-specific. A single LAB strain can function as both a starter and a protective culture at the same time.

Since the protective effect of lactic acid bacteria usually depends on acid production, adding them to a food product that is not intended to ferment inevitably leads to some sensory changes: a sour taste, flavor compounds, gas formation, or changes in texture. A truly “invisible” protective culture is therefore only possible if the inhibitory effect is based on low concentrations of specific metabolites (e.g., a bacteriocin) that act at levels far below the sensory or acidification threshold, or if the strain’s metabolism is restricted by low sugar availability and refrigeration temperatures [13,14,30,31,32,33].

Protective cultures include specific strains of lactic acid bacteria (LAB), such as *Lactococcus* (*Lc.*) *lactis*, *Latilactobacillus* (*Lt.*) *sakei*, *Lactiplantibacillus* (*Lp.*) *plantarum*, *Lacticaseibacillus* (*Ls.*) *paracasei*, *Ls. rhamnosus*, and *Levilactobacillus* (*Lv.*)*. brevis*, as well as *Enterococcus* (*Ec.*) *faecium*, *Carnobacterium*, *Bifidobacterium*, and *Propionibacterium*. The use of protective cultures has proven to be an important clean-label strategy that offers an alternative to E-number preservatives [4,14,34,35,36].

Protective cultures are also used in ready meals to naturally counteract microbial spoilage and preserve freshness. Spraying protective cultures onto individual sausage slices between cutting and packaging, a critical moment for microbial recontamination, offers innovative new applications [37].

Customized protective or starter cultures and their bacteriocins are used to reduce nitrite concentrations in sausage products. Some strains are also reported to have health-promoting properties, including probiotic potential (Section 3.2) [38]. The most commonly used starter and protective cultures in food are provided in Figure 1 [39].

### 3.1. Research Focus on Protective Cultures for Cooked Meat Products

A comprehensive review of the literature on protective cultures in meat products identified 30 different potential cultures, either alone or in strain combinations that were specifically selected for their antimicrobial properties. Twenty-six of these strains (87%) demonstrated a significant inhibitory effect on target organisms (Table 1). Most studies prioritized the efficacy of protective cultures against *L. monocytogenes*, the most critical pathogen in ready-to-eat (RTE) meat products. Several investigations have employed non-pathogenic surrogate strains to model *L. monocytogenes* behavior. *L. innocua* and *L. ivanovii* have been used due to their physiological similarities to *L. monocytogenes* and because they pose no direct human health risk [40,41,42].

**Figure 2:**
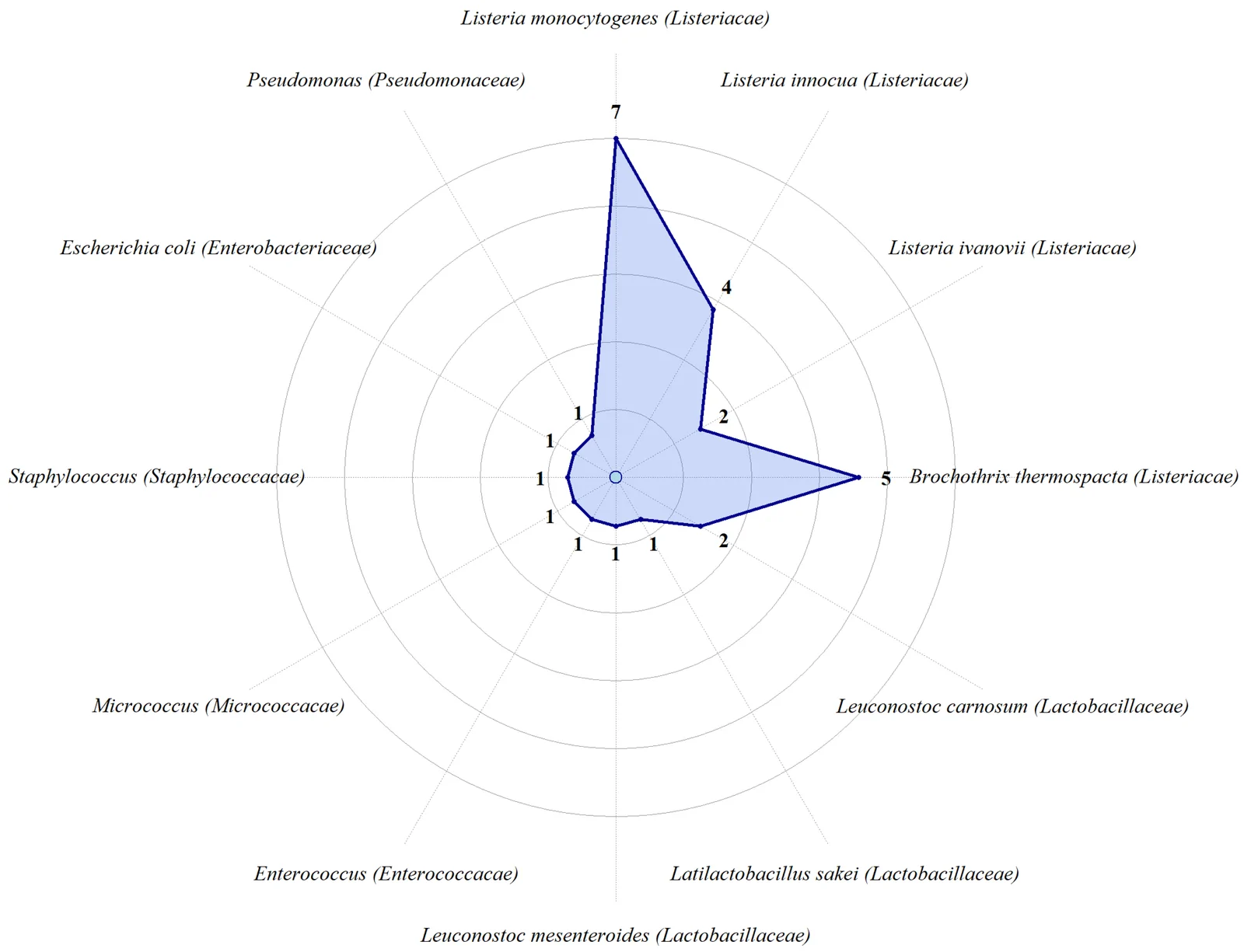
The target organisms used in scientific studies to demonstrate the effectiveness of protective cultures in cooked meat products.

While extensive research has been conducted on pathogenic microorganisms, particularly *L. monocytogenes*, the available literature on spoilage microorganisms remains limited and focuses primarily on *B. thermosphacta*, a major spoilage organism in chilled, non-heat-processed meat products. This gap is even more pronounced for cooked and ready-to-eat meat products, where the dominant spoilage association consists of LAB themselves (*Lactobacillus/Latilactobacillus*, *Leuconostoc*, *Weissella*, or *Carnobacterium*). Since protective LAB cultures share the same ecological niche, substrate requirements, and acid-based metabolism as these spoilage LAB, they cannot selectively suppress them. The bioprotection concept is therefore largely inapplicable against LAB spoilage, which likely explains the scarcity of studies in this product category. Control of LAB spoilage in cooked meats consequently relies on physicochemical hurdles and hygienic measures rather than on microbial competition (Figure 2, Table 1).

**Table 1:** Protective cultures tested against target organisms in studies on cooked meat products, in relation to packaging conditions (vacuum or modified atmosphere packaging). An “X” indicates that the respective protective culture was applied against the listed target organism under the respective packaging condition. Sources: [8,9,10,11,12,13,14,15,16,17,18,19,20,21,22]. Abbreviations: VAC, vacuum; MAP, modified atmosphere packaging.

Target Microorganism(s)														
Protective Cultures	*L. innocua*	*L. ivanovii*	*L. monocytogenes*	*B. thermosphacta*	*Enterococcus* spp.	*E. coli*	*C. perfringens*	*Leuc. mesenteroides*	*Leuc. carnosum*	*Lt. sakei*	*Micrococcus*	*Pseudomonas*	*Staphylococcus*	*Source*
**Vacuum**														
*C. maltaromaticum* CM_B824; B827; B829	X													[40]
*Ec. faecium* CTC492									X	X				[43]
*Companilactobacillus alimentarius* BJ-33			X	X				X	X					[44]
*Leuc. carnosum* 4010			X											[45]
*Lt. curvatus* ACU-1	X													[46]
*Lc. lactis* BB24			X	X				X	X					[44]
*Lc. lactis* G18			X	X				X	X					[44]
*Lc. lactis* UW1			X	X				X	X					[44]
*Lc. lactis* subsp. *lactis* L201			X	X				X	X					[44]
*Leuc. mesenteroides* L124			X	X				X	X					[47]
*Lp. plantarum* ALC			X	X				X	X					[44]
*Lp plantarum* LP5			X	X				X	X					[48]
*Lt. sakei* 148			X	X				X	X					[44]
*Lt. sakei* Lb 706			X	X				X	X					[44]
*Lt. sakei* subsp. *carnosus* GERT17			X	X				X	X					[44]
*Lt. sakei* C2			X											[49]
*Lt. sakei* CTC494									X	X				[42]
*Lt. sakei* L2512	X	X												[42]
*Lt. sakei* SAGA 777			X	X				X	X					[44]
*Lt. sakei* TH1			X			X								[50]
*P. acidilactici* H (wild typ of JBL1095)			X											[51]
*P. acidilactici* LMQS 154.1, *P. pentosaceus* LMQS 331.3			X	X										[52]
*P. acidilactici* PA-2			X	X				X	X					[44]
*S. xylosus* DD-34				X							X	X	X	[53]
**Modified Atmosphere Packaging and Vacuum**														
*Lt. curvatus* L442					X	X								[47]
*Lc. lactis* DSM 20729, *P.acidilactici* LMQS 154.1				X	X									[52]
*P. acidilactici* PAC JC 1-23			X											[54]
**Modified Atmosphere Packaging**														
*Leuc. carnosum* 4010	X	X												[41]

With the exception of two studies focusing on *Pseudomonas* and *Escherichia coli* (*E. coli*), the target organisms were exclusively Gram-positive bacteria. One potential explanation for this trend is the displacement of Gram-negative bacteria, such as *Pseudomonas*, by Gram-positive bacteria, such as *Latilactobacillus*, during the product’s shelf life. Gram-positive bacteria are known to exhibit greater tolerance to lower water activity and/or lower pH [55]. Additionally, recent studies have identified yeasts as contributors to spoilage, as they were inhibited but not fully eliminated in cooked ham by protective cultures [48].

The dosage of protective cultures is critical and must be tailored to the timing of application (pre- vs. post-heating) and the specific challenges of the meat product (e.g., pH, water activity, competing microbiota). In studies where *Lc. lactis* DSM 20729, *Pediococcus* (*P.*) *acidilactici* LMQS 154.1, *P. pentosaceus* DSM 331.3, and *Lt. curvatus* ACU-1 failed to achieve the desired reduction, this may be attributed to the low initial concentration of the protective cultures used [52]. The concentration of the protective cultures used should be between 10^5^ log and 10^7^ CFU/g when administered after heating and between 10^8^ and 10^10^ CFU/g when administered before heating [41,50,53].

A review of studies on protective cultures revealed that Berry et al. [54], Jacobsen et al. [41], and Kotzekidou and Bloukas [53] examined their effects prior to heating. In the remaining studies, the cultures were applied exclusively to the surface after heating (Table 1 and Figure 2).

Protective cultures can be applied to sausage meat prior to heating during emulsification, the brine injection, or the tumbling of the ham. Jacobsen et al. [41] argue that incorporating protective cultures prior to heating is not only advantageous from a process engineering standpoint but also effective in combating *Listeria*. An innovative method for adding protective cultures before heat treatment is encapsulation. A variety of stimuli—such as temperature, pH, mechanical stress, and osmotic pressure fluctuations—have been shown to trigger the release of encapsulated microorganisms. Nevertheless, the efficacy of this approach in cooked meat products remain unproven and has, to date, only been demonstrated in high-pressure-treated products [56,57,58,59].

The efficacy of protective cultures can be enhanced by combining them with bacteriocins or through the use of multiple protective cultures together in products like cooked ham. This synergistic effect has been demonstrated in both cooked meat products and vacuum-packed lamb [33,49]. When protective cultures and bacteriocins are used in tandem, the bacteriocin initially inhibits the growth of undesirable bacteria, providing a window for the protective culture to reach a sufficient cell density to effectively suppress the target bacteria [60].

Certain microbial communities—comprising environmental, spoilage, and pathogenic organisms—are increasingly adapting to production environments, posing a risk of contaminating food and food ingredients. This adaptation has contributed to a notable rise in the prevalence of Shiga toxin-producing *E. coli* (STEC), *Listeria monocytogenes*, *Salmonella*, and *Campylobacter* across several European countries (ECDC, https://www.ecdc.europa.eu/en/food-and-waterborne-diseases-and-zoonoses/surveillance-and-disease-data, accessed on 5 November 2026) [61,62]. Therefore, epidemiological data should be considered when selecting target pathogenic strains. In particular, *L. monocytogenes* strains of the clonal complex (CC) of lineage II are particularly common in food. For example, CC 121 and CC9 are known to be persistent in food processing environments [63]. Additionally, data obtained from a single reference strain cannot be generalized to the entire species or even to a specific genetic lineage. Consequently, efficacy tests should always include multiple strains, ideally incorporating field isolates to ensure broader applicability [64].

### 3.2. Biological Safety of Protective Cultures

LAB and their bacteriocins are generally recognized as safe (GRAS) and offer beneficial properties, including antimicrobial, antioxidant, antihypertensive, immunomodulatory, anti-inflammatory, and potential anticancer effects [12,65]. However, careful evaluation of health-related biosafety factors remains essential when selecting them as protective cultures [12,65].

From a legal perspective, only microbial strains that have been rigorously tested and approved by the European Food Safety Authority (EFSA, https://www.efsa.europa.eu/en/topics/topic/qualified-presumption-safety-qps, accessed on 5 November 2026) or classified as “Generally Recognized as Safe” by the US Food and Drug Administration (FDA) (GRAS, https://www.fda.gov/food/food-ingredients-packaging, accessed on 5 November 2026) [29,66] may be used as starter or protective cultures in food production.

These approvals guarantee the safety, characterization, and effectiveness of the strains for improving food quality, safety, and preservation (Supplementary Table S1). Regarding product labeling, EU legislation (Regulation (EU) No. 1169/2011) exempts ingredients from being listed if they do not perform a technological function in the final product—such as when they are reduced to natural levels during thermal processing [67]. However, if the cultures remain active in the final product (as is typically the case with protective cultures), they must be declared on the label.

Additionally, Regulation (EC) No. 852/2004 mandates the verification of food safety measures [68].

Bacteriocin-producing microorganisms, including LAB and other taxa, along with their bacteriocins, show great potential as protective cultures by providing antimicrobial, probiotic, and health-promoting effects. However, the implementation of these technologies necessitates a meticulous evaluation of biosafety concerns. LAB have been observed to harbor antibiotic resistance genes, which have the potential to transfer to pathogens. Furthermore, certain strains of LAB have been found to produce biogenic amines in fermented foods. To ensure safety, strain-specific assessments must be conducted prior to their use. These assessments include testing for the minimum inhibitory concentrations (MICs) of key antibiotics and screening for biogenic amine production and virulence factors [69,70,71]. If these risks, costs, and technological challenges—such as maintaining the stability and activity of postbiotic compounds (consisting of metabolic byproducts, cell components, or secreted factors that provide health benefits) during food processing—can be addressed, LAB have the potential to enhance food safety, extend shelf life, and improve nutritional and health-promoting properties. The establishment of clear regulatory guidelines could further facilitate their safe and effective integration into food production [12,35,72].

### 3.3. Bacteriocin Application in Cooked Meat Products and Limitations

**Figure 3:**
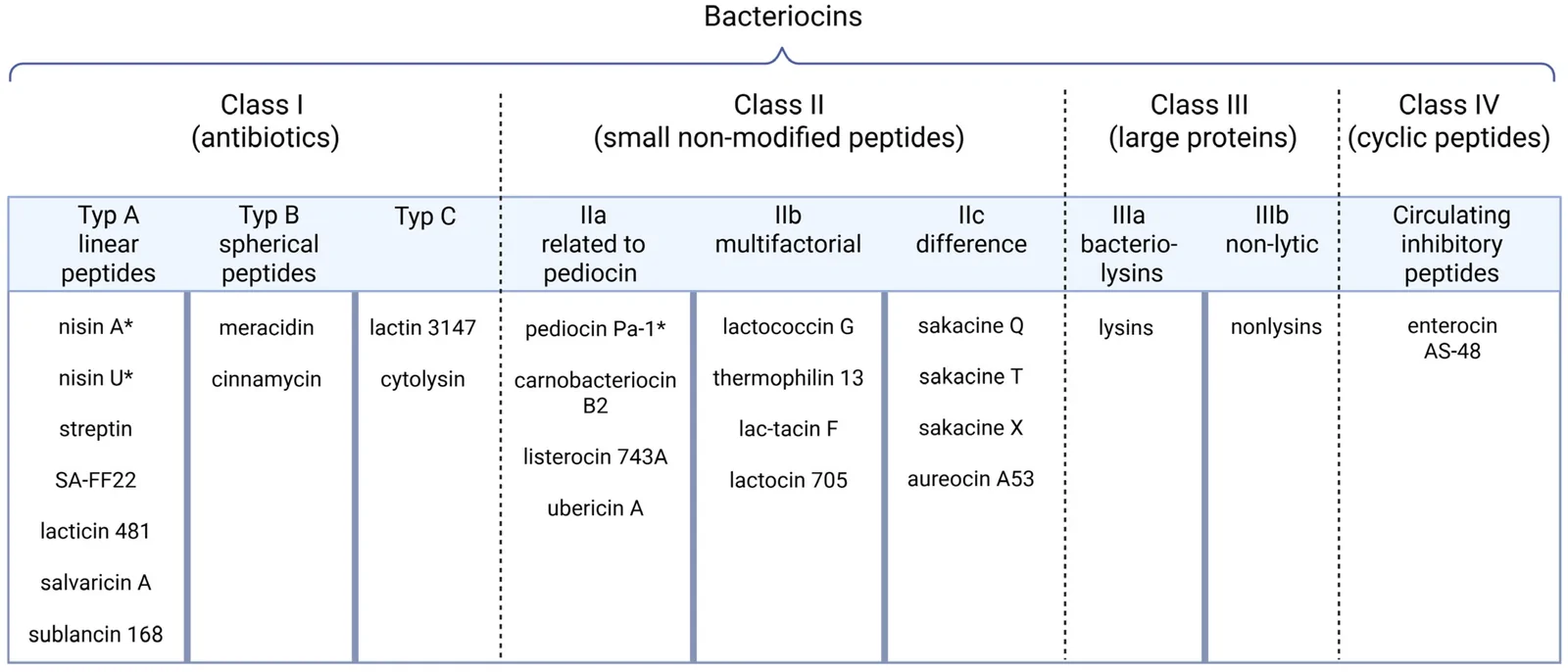
A list of bacteriocin classes, their categories and types, and exemplary bacteriocins per category, modified from [39]. Approved bacteriocins are marked with an *. Created in BioRender. Hennes, T. (2026) https://BioRender.com/m10354z, accessed on 5 November 2026.

Bacteriocins are classified into four classes and nine subcategories. A significant distinction is made between post-translationally modified peptides (class Ia) and unmodified peptides (class II) [65,73] (Figure 3).

Table 2 provides a comprehensive overview of the bacteriocins utilized in research on meat products. The most prevalent strains were those capable of producing sakacin, followed by those capable of producing pediocin and enterocin (Table 2).

Bacteriocin AS-48, produced by *Ec. faecalis* subsp. *liquefaciens*, has been identified as an effective solution for addressing spoilage LAB, *B. thermosphacta*, and *S. carnosus* [78,89]. The application of a bacteriocin mixture produced by *Lt. curvatus*, *Lc. lactis*, *P. acidilactici*, and *Enterococci* appeared to be promising, as evidenced by the reduction in *L. monocytogenes* on hot dog sausages [90]. It has been demonstrated that Micocin^®^ (a mixture of bacteriocins produced by *C. maltaromaticum* CB1) is effective against *Clostridium botulinum* (*Cl.*) and *L. monocytogenes* [89].

Gao et al. [49] demonstrated that the combination of bacteriocin and a protective culture (sakacin C and *Lt. sakei* C2) exhibited a greater degree of effectiveness against *L. monocytogenes* than either the protective culture or the bacteriocin alone in vacuum-cooked ham.

As with protective cultures, the concentration of bacteriocins must be carefully determined to ensure effectiveness. For example, a study found that nisin concentrations of 12.5 mg/kg failed to inhibit *Listeria* growth, whereas 32 mg/kg demonstrated an inhibitory effect [91]. The established correlation between bacteriocin production and optimal cell growth suggests the possibility that not all bacteriocin-producing strains present in specific food matrices actively secrete bacteriocins. The optimal conditions for the formation of bacteriocins for product preservation are contingent upon environmental factors such as temperature, salt concentration, pH, and packaging atmosphere [92]. Technological processing steps or other recipe components, such as brine, have been demonstrated to exert antagonistic effects [91].

The effectiveness of bacteriocins is also influenced by the food matrix. For instance, Danielski et al. [40] found that *C. maltaromaticum* produced only minimal bacteriocin levels in cooked ham, likely due to limitations imposed by the food matrix. Similarly, the antimicrobial activity of *Ec. faecium* bacteriocins can vary significantly depending on the food type, processing conditions, and composition.

While the efficacy of *Ec. faecium* bacteriocins has been well documented in cheeses and fermented soybeans, their performance in processed meats and other complex foods has proven less consistent [93].

The bioconservative effect of bacteriocins in meat and meat products is currently only partially exploited commercially, with nisin and pediocin PA-1 being commercially available for food preservation. The prevailing legal framework in both the US and EU presently permits the utilization of nisin (E 234) exclusively as a pure bacteriocin (Food & Drug Administration (FDA), https://www.law.cornell.edu/cfr/text/21/184.1538, accessed on 5 November 2026) [94,95,96,97]. In the United States, certain pediocin-based preparations have received regulatory approval (FDA, https://downloads.regulations.gov/FDA-2025-N-1927-0046/attachment_1.pdf) accessed on 5 November 2026 [98].

Nisin resistance in *L. monocytogenes* with increasing NaCl exposure has been well documented and is important to note. In addition to environmental factors, the genetic background also plays a decisive role [99,100]. The resistance mechanisms described are mediated by efflux pumps and regulatory systems (VirAB, VirSR, and the multicomponent system AnrAB), and such shared mechanisms can lead to cross-resistance against nisin, ciprofloxacin, ethidium bromide, bacitracin, cephalosporins, and benzalkonium chloride [101,102]. Beyond genetic co-resistance, exposure to benzalkonium chloride—a disinfectant commonly used in food processing—can further promote co-selection.

This is supported by the frequent colocalization of genes for benzalkonium chloride resistance (e.g., *bcrABC*, *emrC*) with genes that confer acquired antibiotic resistance [103,104,105].

The gadD3 gene (part of the GAD system) and mutations in *mpt/manR* contribute to resistance against nisin and class IIa bacteriocins. Resistance also depends on the clonal complex (CC), with SSI-1 (in CC3, CC5, CC7, CC9) enhancing resistance under salt stress [100]. Additional mutations (e.g., RsbU G77S, PBPB3 V240F) in CC7 and CC121 further increase resistance and may enhance virulence [93,94]. CC2, CC3, CC6, CC7, CC14, CC199, and CC403 exhibit particularly high resistance, underscoring the role of the genetic background [100,106].

However, the inherent resistance of *L. monocytogenes* to nisin significantly limits its effectiveness, making it necessary to use it in combination with other additives or technological measures [94]. Cross-resistance mechanisms among bacteriocins do occur as strains that are resistant to nisin, and often also resistant to class IIa bacteriocins (e.g., pediocin) [107].

In order to achieve the full potential of bacteriocins, several challenges must be overcome. These objectives include the optimization of methods for identifying bacteriocin genic strains and the validation of their activity in situ, as well as the coordination of political and regulatory processes to establish harmonized frameworks that facilitate the appropriate and efficient utilization of bacteriocin-producing strains and bacteriocins [36,108]. Presently, bacteriocins are required to meet specific approval criteria, encompassing proper identification, characterization of their chemical composition, and safety assessment, as well as an indication of the recommended application concentration. Despite the challenges associated with large-scale application, bacteriocins have significant potential for improving food safety and extending shelf life [89].

### 3.4. Application of Protective Cultures in PBMAs

Chen et al. [109], Lindberg et al. [110], and Xie et al. [111] have conducted systematic reviews examining the environmental, processing, nutritional, health, and future perspectives of PBMAs, while Munialo et al. [112] provided a comprehensive overview of meat substitutes, including protein sources, quality characteristics, analytical methods, and consumer perceptions. However, the application of protective cultures in PBMAs has yet to be explored in scientific detail. Key challenges and future directions involve selecting the right protective culture, ensuring its feasibility for industrial implementation, and combining these cultures with emerging protein sources to create safe, high-quality food products [113].

A promising approach in extruded PBMAs involved a combination of 1% chitosan, 2.5% tea polyphenols, and 0.04% nisin as an alternative to conventional preservatives, which had a shelf-life four times longer than the untreated control samples [114].

In the field of flaxseed oil cake-based plant milk alternatives for the development of innovative probiotic beverages, the incorporation of *Ls. rhamnosus* in combination with sucrose-driven acidification has been shown to effectively inhibit pathogenic microorganisms such as *Bacillus cereus* (*Bc.*) [115]. *Ls. rhamnosus* has been demonstrated to exert a protective effect against *E. coli* 0157:H7 in the context of fermented raw sausages [116].

Elhalis et al. [117] provided a comprehensive overview of the pivotal factors influencing the development of protective cultures in PBMAs. Key aspects that should be considered include the ability to displace the natural microflora, the detoxification of mycotoxins and biogenic amines, cost-effectiveness, and processing and storage stability. The cultures must demonstrate resilience to rigorous processing techniques, such as extrusion, while enhancing flavor, texture, and safety attributes.

Starter and protective cultures often overlap in terms of their species and functions, so factors such as adaptability, detoxification, and stability apply to both. An example is tofu, a traditional soy-based meat alternative in which nisin, in combination with protective cultures (*Ec. faecium* BFE 900-6a or *Lc. lactis* BFE 902), completely inhibit *L. monocytogenes* [118]. Nisin initially suppresses *Listeria* until the protective cultures reach a sufficient density to take over long-term inhibition [60]. This approach demonstrates how hurdle technology—the combination of bacteriocins and protective cultures—can enhance the safety of plant-based meat alternatives. The principle could be applied to extruded or moisture-rich plant-based products to extend shelf life and improve safety without relying on synthetic preservatives.

## 4. Process Differences Between Cooked Meat Products and PBMAs

**Figure 4:**
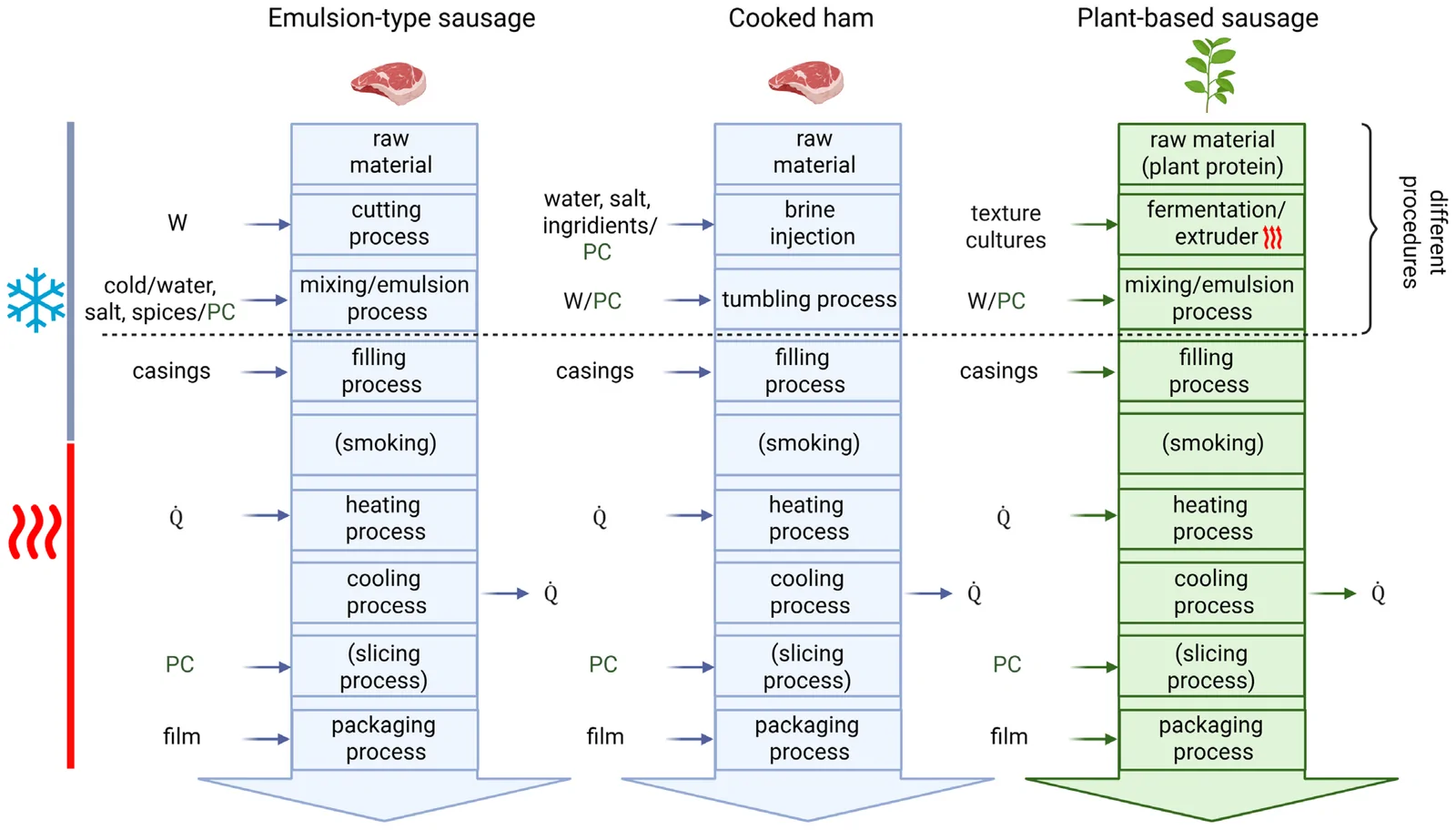
Flow diagrams illustrating the manufacturing processes for “emulsion-type sausage,” “cooked ham,” and “plant-based sausage.” Abbreviations: PC: protective cultures; W: work; Q: heat flux. Created in BioRender. Hennes, T. (2026) https://BioRender.com/757di7x, accessed on 5 November 2026.

To select the optimal protective culture, the manufacturing process must first be described (Figure 4), as well as the process step in which the culture is to be used (Section 3.1). The type of packaging is important, as products are sold either in slices or whole, and either vacuum-packed or under a modified atmosphere, depending on market requirements. The added protective culture must have an advantage over competing flora in the respective packaging [65]. When using protective cultures, it is essential to check their compatibility with starter cultures used at the same time (e.g., *S. xylosus* and *Lt. sakei*) to avoid unintended inhibition. Pilot-scale trials or in vitro co-culture tests are recommended prior to large-scale application [119].

### 4.1. Emulsion-Type Sausage

Cooked sausages are food products made from a mixture of meat, fat, water, salt, spices, and additives, with fat distributed within a homogenized protein matrix [120]. They can be classified based on raw materials, additives, mincing degree, size, and consumption method [121].

The production process typically involves grinding ingredients into a fine mixture (sausage meat or emulsion), facilitated by the breakdown of sarcomeres and activation of muscle proteins like myosin and actin, which act as emulsifiers. Salt (NaCl) enhances protein unfolding by increasing electrostatic repulsion, allowing proteins to stabilize lipid and aqueous phases [122,123]. The mixture is then stuffed into natural or artificial casings (e.g., collagen, cellulose, plastic, or co-extruded alginate-collagen hybrids) [124].

Heat treatment (>65 °C) denatures proteins, converting the emulsion into a solid gel [125,126]. After heating, cooling stabilizes the product microbiologically [127] (Figure 4).

### 4.2. Cooked Ham

In traditional cooked ham production, whole legs are used, though modern processes may employ alternative cuts or smaller meat pieces [128]. The process begins with brine injection (containing sodium chloride, nitrite, spices, phosphates, salts, ascorbate, and sugar) using a needle injector [129]. This is followed by tumbling, where meat is lifted and dropped in a rotating drum, converting kinetic energy into potential energy. The combination of drop height and force improves tenderness, water-holding capacity, and yield [130,131]. Tumbling also disrupts meat cells, releasing myofibrillar proteins that act as natural binders [132], while LAB counts increase by up to 3 log CFU/g [133].

The ham is then heated to a core temperature of 70 ± 5 °C, reducing bacterial populations by ~50%, though heat-stable species may reappear after a latency phase [134,135]. As with cooked sausages, rapid cooling follows heating to stabilize microorganisms and prevent regrowth [127].

### 4.3. Plant-Based Sausage Processing

Plant-based proteins that share a structural similarity with myofibrillar meat proteins are crucial for creating meat-like textures in meat alternatives (Supplementary Table S2). Common sources include soy, peas, chickpeas, lentils, and mushrooms, which are used in textured (e.g., TVP, extrudates) or non-textured forms (e.g., concentrates) to mimic muscle fibers [123].

Vegetable oils (rapeseed, coconut, palm) replace animal fats, while thickening agents (carrageenan, xanthan gum, methylcellulose) stabilize emulsions and improve moisture content and mouthfeel [118,135,136]. The addition of ingredients such as wheat gluten or pectin has been shown to result in a fibrous texture [137]. Edible mushrooms (e.g., Pleurotus ostreatus, Lentinula edodes) offer umami flavor and fibrous texture. Genetic modifications (e.g., enhanced protein expression or reduced bitterness) could optimize their scalability and sensory profile for meat substitutes [118,138,139]. Research initiatives, nascent businesses and established corporations are engaged in the development of alternative protein sources as meat substitutes on a microbiological basis. These include bacteria, microalgae, fungi, and yeasts [140].

The extrusion of pea proteins at a high moisture content, as well as lactic acid fermentation, reduces the pea-like odor caused by n-hexanal [141, 142,143]. Shear cell technology can also achieve a meat-like structure under similar critical processing variables [144,145,146].

The fermentation of PBMAs improves their nutritional value and organoleptic properties, such as by reducing the bitterness and increasing the juiciness of soy proteins [147,148]. *Bc. subtilis* improves the fermentation of extruded plant-based proteins, thereby enhancing the product’s texture and nutritional value [141]. Off-flavors that may occur are associated with an earthy, bitter, astringent, or bean-like taste. To counteract these deviations, the fermentation of plant matrices with LABs is employed [149].

*Lactobacillus* (*Lb.*) *delbrueckii* subsp. *bulgaricus*, *Streptococcus thermophilus*, *Lp. plantarum*, and *Limosilactobacillus fermentum* play a crucial role in improving plant-based ingredients like field beans, hemp, and soybeans by addressing both anti-nutritional compounds and off-flavors. *Lb. delbrueckii* subsp. *bulgaricus*, *Lp. plantarum* and the animal-derived *Lb. acidophilus* significantly reduce hexanal and hexanoic acid, which are responsible for undesirable grassy or rancid notes, thereby enhancing the overall flavor profile of plant extracts [150]. Although the enzymatic repertoire of LAB is generally limited, their metabolism reduces several antinutritional factors in fermented foods. Phytates, which chelate iron and zinc, are degraded mainly indirectly via acid activation of endogenous plant phytases—with only weak, strain-dependent dephosphorylation by intracellular nonspecific acid phosphatases of selected LAB strains. Meanwhile, oxalates and raffinose-family oligosaccharides are also degraded, and bitter, astringent phenolic compounds and saponins are modified. Additionally, enzymatic activities such as α-galactosidase, phytase, amylases, lipases, and proteases enhance nutrient bioavailability, digestibility, and the release of bioactive compounds [151,152].

The subsequent filling of the PBMAs can be performed using a conventional sausage-filling machine into a casing made of cellulose, plastic, or alginate [124,142]. The heating process leads to denaturation and changes in protein folding [125]. To prevent the germination of heat-stable spore-forming bacteria such as *Bacillus* spp. and *Clostridium* spp. after heating, an additional heating step for decontamination is often required [153,154]. Heat treatment at 120 ± 2 °C for 30 min has been demonstrated to yield comparable microbial inactivation and structural modifications in both animal-derived and plant-based food matrices, ensuring effective pathogen reduction and product stability [155]. However, the development of thermal processing protocols for novel plant-based products necessitates product-specific optimization to address their distinct physicochemical and sensory attributes.

A critical aspect of this process is the establishment of customized thermal profiles that maintain functional properties such as protein denaturation, moisture retention, and texture, while accounting for variations in protein sources and lipid substitutes. Furthermore, post-thermal pasteurization in final packaging, particularly for high-moisture products like plant-based deli slices, is imperative to ensure extended shelf life without compromising organoleptic or nutritional quality [156].

The optimization of protective culture incorporation, before or after heating, is important to prevent thermal inactivation or sublethal injury, which could compromise their competitive efficacy. Moreover, rapid post-thermal cooling is a critical step in minimizing microbial proliferation and preserving structural integrity, in accordance with conventional meat processing standards [153,154].

### 4.4. Plant-Based Products Available on the Market

Over 1500 companies worldwide now produce plant-based meat alternatives on a large scale [157, 158]. Key protein sources include soy, pea, lentil, chickpea, wheat gluten, rice, potato, mycoprotein, algae, sunflower, and rapeseed (see Supplementary Table S2) [137,138,148,157,158,159,160,161,162,163]. The variety of raw materials is important from a microbiological standpoint because the protein source, moisture content, and processing method determine the product’s water activity, pH, and associated microbial hazards—as well as the conditions that a protective culture must satisfy.

## 5. Microbiology of PBMAs

Despite the multiple advances of plant-based products, critical knowledge gaps remain, particularly regarding the microbial safety and spoilage potential of these newer protein sources. The initially low bacterial count in PBMAs is primarily attributable to the extrusion process of the raw materials [164,165]. The accelerated growth can be attributed to the specific physicochemical properties that currently favor the survival and proliferation of microorganisms in plant-based products. These include, in particular, a nearly neutral pH value (which is traditionally somewhat lower in sausage products), a high-water activity (a_W_), and a high protein and moisture content [166].

The limited research on their microbial flora is partly due to their recent market introduction, leaving significant uncertainties regarding resistance to pathogens, spore-forming bacteria, and optimal inactivation methods [154,167].

To address these challenges, targeted microbial characterization is essential, focusing not only on the total plate count but also on spore-forming bacteria and heat-resistant pathogens that can compromise product safety and shelf life [168]. The development of tailored processing techniques—such as optimized heat treatments, fermentation protocols, or natural preservation systems—will be crucial to ensuring the safety, stability, and consumer acceptance of these innovative protein sources.

### 5.1. Microbiological Diversity of PBMAs

The level of microbial contamination in plant-based protein raw materials—even among raw materials of the same type—can vary significantly, influenced by a complex interplay of various factors along the supply and processing chain. These factors include geographic origin, environmental conditions (e.g., temperature, humidity, and precipitation during cultivation), agricultural practices (e.g., use of pesticides and fertilizers), harvesting techniques, storage conditions, transport logistics, and subsequent processing methods [168]. While plant-based protein raw materials are generally well-suited for PBMAs due to their functional and nutritional properties, their microbial profiles reveal both opportunities and challenges. *Enterobacteriaceae* (e.g., *E. coli*, *Salmonella* spp., *Klebsiella* spp., *Enterobacter* spp.) are frequently present in soil and irrigation water, particularly in organically fertilized fields [154]. Wild animals, livestock, or insects can transmit Enterobacteria to plants via fecal contamination [167]. High humidity or improper drying can promote bacterial growth, particularly in high-moisture ingredients (e.g., pea protein concentrates, soy flour) [153]. Enterobacteria can be introduced by harvesting machinery and also during processing, such as grinding, extrusion, or mixing, if the equipment or processing environment is not adequately disinfected. Poor-quality process water (e.g., for washing or rehydration) can lead to recontamination (ILSI, https://ilsi.eu/publication/the-enterobacteriaceae-and-their-significance-to-the-food-industry/, accessed on 5 November 2026) [168,169].

*Salmonella*, as a foodborne pathogen, is relevant in the risk analysis of PBMAs because both contaminated raw materials play a role and the pathogen exhibits high tenacity and survivability in low-moisture foods (LMFs). Recalls occurred for plant-based chicken meat alternatives, soy-based meat substitutes, cashew cheese, and wild berry diet supplements (https://www.fsis.usda.gov/recalls; https://www.fda.gov/food/outbreaks-foodborne-illness/outbreak-investigation-salmonella-jules-cashew-brie-april-2021; https://www.fda.gov/safety/recalls-market-withdrawals-safety-alerts/superfoods-inc-dba-live-it-recalls-live-it-super-greens-because-possible-health-risk accessed on 5 November 2026) [170,171,172].

*Salmonella* and *Cronobacter sakazakii* exhibited similar growth and survival rates when compared in meat juices (from plant- and animal-based products). Furthermore, *L. monocytogenes* showed a higher survival rate in plant-based meat substitutes [173]. *L. monocytogenes*, persistent in nature and food processing environments, can grow at refrigeration temperatures, posing a risk in RTE PBMAs. Recalls occurred with vegan beverages, plant based meat alternatives, vegan organic cheese, and a foie gras alternative (https://www.canada.ca/en/public-health/services/public-health-notices/2024/outbreak-listeria-infections-recalled-refrigerated-plant-based-beverages.html; https://www.sciensano.be/en/biblio/bsfm-2023-L-monocytogenes-plant-based-foods-insights-a-recent-foodborne-outbreak-linked-vegan#:~:text=Due%20to%20the%20growing%20demand,monocytogenes%20in%20plant%2Dbased%20foods; https://food.ec.europa.eu/food-safety/rasff_en accessed on 5 November 2026) [174,175,176]. A 2022–2024 European listeriosis outbreak was linked to contaminated plant-based, fermented vegan cheeses made from cashews and coconut milk [177].

Gram-positive bacteria are usually found in low concentrations (<100 CFU/g), particularly in extruded raw materials [178]. However, the microbial diversity of plant proteins is often dominated by spore-forming bacteria (e.g., *Bc. subtilis*, *Bc. amyloliquefaciens*, and *Bc. licheniformis* or *Clostridium* spp.), which are known for their heat resistance and ability to survive conventional thermal processing [168]. A high prevalence of *Cl. botulinum* was found in vegetarian sausages, suggesting inadequate heat treatment and a link to the raw materials [179]. *Bc. cereus* was detected in over 80% of PBMAs in the Czech Republic [180].

Therefore, it is important to identify protective cultures in combination with other technological barriers against the variety of spore-forming microorganisms that dominate plant-based products. As endospores can tolerate acid-based mechanisms, protective cultures should be considered part of a series of barriers rather than a standalone measure [181]. Typical extrusion parameters (120–160 °C with a short residence time) result in only a 2–3 log reduction in spores. Hence, protective cultures should ideally be added after extrusion. Furthermore, dense protein gels can limit the diffusion of bacteriocins. Validating such combinations of multiple barriers against the growth spore in plant-based protein matrices is a key area of ongoing research.

While often beneficial for fermentation and preservation, LAB can also contribute to unintended acidification or flavor changes if handled improperly [151]. Enterococci and other facultative anaerobic bacteria can survive in oxygen-deprived environments and could pose potential safety risks if pathogenic strains are present [167], and *Leuconostoc mesenteroides* (*Leuc.*) and *Lt. sakei*, *Ec. faecium*, and *C. divergens* appear to be the predominant bacteria in PBMAs [167,182,183]. A study conducted in Finland showed that *Leuc. gelidum*, *Leuc. gasicomitatum*, and *Leuc. mesenteroides* were the predominant microorganisms in a vegetarian carrot sausage causing spoilage [184].

Plant proteins can also harbor a range of other microorganisms, including yeasts and molds, which can proliferate under high humidity or improper storage conditions, leading to spoilage, off-flavors, or the formation of mycotoxins (e.g., species of the genera *Aspergillus*, *Penicillium*, and *Fusarium*) [152,185].

The presence of these diverse microbial populations requires a multi-faceted approach to microbial control, including the following methods: Optimized thermal and non-thermal processing methods (e.g., high-pressure treatment, pulsed electric fields, or cold plasma) can inactivate heat-resistant microbes or spores [186]. Fermentation or biopreservation strategies that use protective cultures (e.g., *Lp. plantarum*) can suppress spoilage microorganisms and extend shelf life. The presence of molds, yeasts, and Enterobacteriaceae in the final product is largely attributable to inadequate process hygiene during the heating process and less so to the quality of the raw material; thus, this can be used as a hygiene indicator [187]. Hygiene and disinfection protocols can be improved throughout the entire supply chain to minimize contamination risks. Regular microbiological monitoring can be implemented to identify and contain emerging microbial threats and ensure compliance with food safety standards. Addressing these microbial challenges is critical to improving the safety, quality, and consumer acceptance of PBMAs [188].

**Figure 5:**
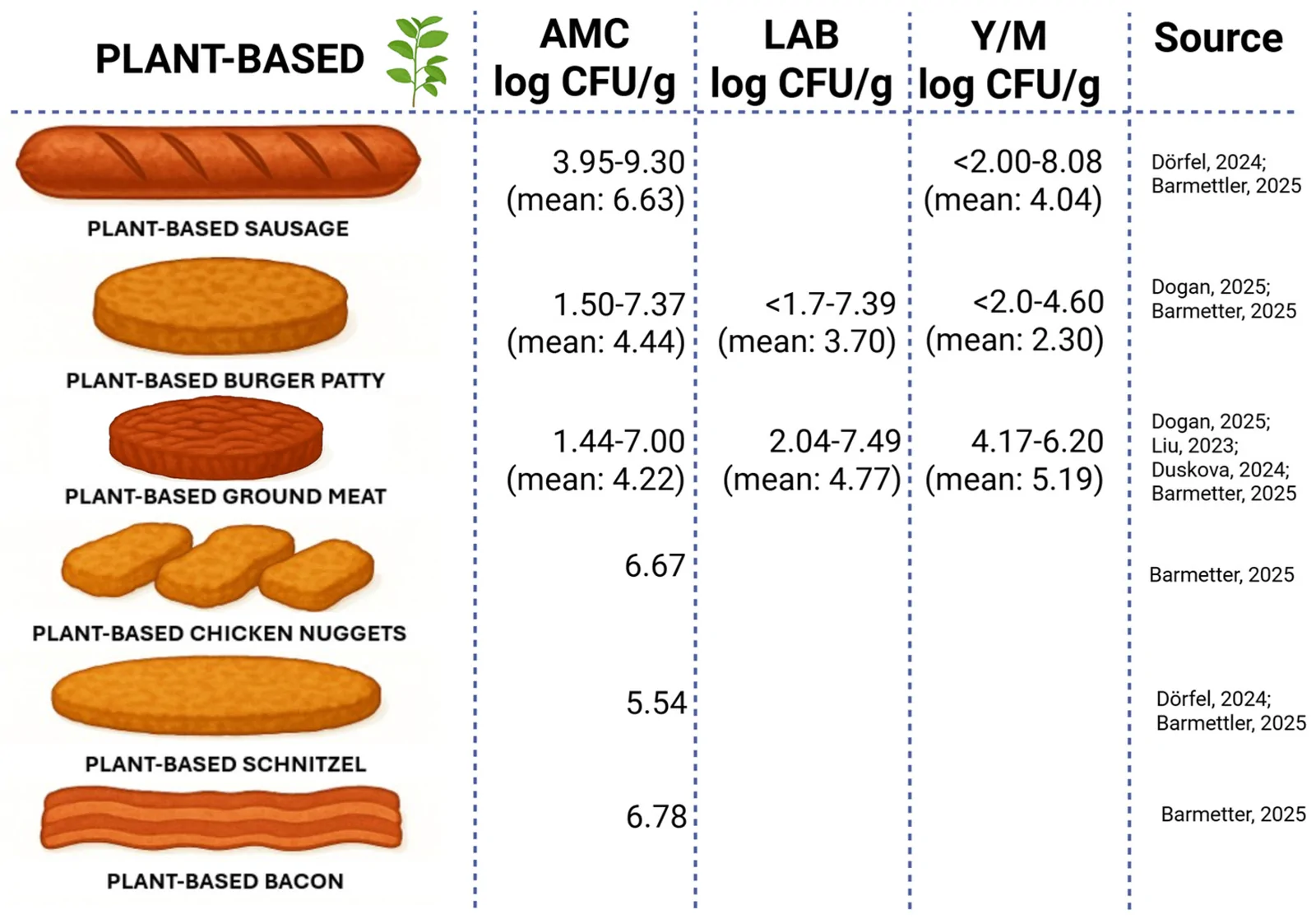
This figure illustrates the total bacterial count of the most popular plant-based meat alternatives, determined in various research studies. Sources: [164,165,180,189,190]. Abbreviations: CFU, colony-forming unit; g, gram; AMC, aerobe mesophile colonies; LAB, lactic acid bacteria; Y/M, yeast/mold. Created in BioRender, incorporating AI-generated elements produced with ChatGPT (OpenAI). Hennes, T. (2026) https://BioRender.com/njg6e9b, accessed on 5 November 2026.

Figure 5 provides an overview of the CFU/g values for total bacterial count, LAB, and yeasts/molds in various plant-based products. It is important to note that the values are derived from different studies and that the microbial counts were determined at different time points depending on the study. Consequently, the reported minimum and maximum values were systematically considered.

### 5.2. Risk of Cross-Contamination During Processing

RTE products, including sausages, are considered particularly susceptible to cross-contamination and recontamination throughout the production and logistics chain [191]. Unlike animal proteins, which are predominantly colonized by LAB, plant-based proteins primarily contain spore-forming bacteria [168]. It is important to note that PBMAs are often produced in facilities that also process animal proteins [159]. Given the differing microbiomes of the raw materials used, it is essential to exercise caution and prevent cross-contamination between animal and plant-based proteins. Since PBMAs promote the growth of microorganisms, it is essential to implement strict measures to prevent the recontamination of these products after heating [166]. Awareness of the potential for recontamination is essential, as it arises from interactions with other product types, equipment that comes into contact with products, surfaces, or personnel. In particular, contact between heat- and non-heat-treated products, as well as inadequate cleaning procedures, pose a risk [191,192]. Given the extensive evidence, it is recommended that production processes for animal and plant-based meat products are strictly separated from one another. As an alternative, there are strong arguments in favor of implementing a staggered production schedule. This should be accompanied by the implementation of comprehensive cleaning and disinfection measures between the two product lines.

### 5.3. Legal Requirements for PBMAs

The regulatory environment for PBMAs remains fragmented and in a state of flux, with only a few standardized guidelines specifically addressing their manufacturing, labeling, or microbiological safety.

Regulation (EU) 2015/2283 (Novel Food Regulation) applies to PBMAs containing novel ingredients (e.g., mycoprotein, algae-based proteins, or new plant extracts) that were not consumed in the EU to a significant degree prior to May 1997. Pre-market authorization and safety assessments are required by the European Food Safety Authority (EFSA). Regulation (EC) No. 1829/2003 (GM Food and Feed) governs PBMAs derived from genetically modified organisms (GMOs) and mandates traceability, labeling, and risk assessment for GMO-derived ingredients (European Commission, 2003) [193].

There are no EU-wide microbiological criteria for PBMAs. This gap is attributed to their recent market introduction and limited empirical data on microbiological risks [169]. General Food Law (Regulation (EC) No. 178/2002) requires the safe marketing of food, but it does not establish any PBMA-specific limits.

Commission Regulation (EC) No. 2073/2005 (microbiological criteria for foodstuffs) applies to ready-to-eat (RTE) foods but does not explicitly address PBMAs.

There are no EU-wide harmonized definitions for the labeling of “vegan” and “vegetarian.” In Germany, the “Guidelines for Vegan and Vegetarian Foods Resembling Foods of Animal Origin” [194] regulate designations and production characteristics and prohibit misleading claims. The Austrian Food Code (A5, Annex 11) establishes labeling requirements for vegan and vegetarian products that refer to animal-based foods [195]. In France, a law (2020-699) has prohibited the use of meat-related terms (e.g., “sausage,” “steak”) for plant-based products since 2024 (Légifrance, https://www.legifrance.gouv.fr/jorf/id/JORFTEXT000049199307 accessesd on 5 November 2026) [196]. There are currently no specific DGHM (German Society for Hygiene and Microbiology) warning values exclusively for vegan or PBMA products. The general warning values for foodstuffs (DGHM, https://www.dghm-richt-warnwerte.de/de accessesd on 5 November 2026) can serve as benchmarks for evaluating the hygienic quality and safety of vegan and plant-based products. These values are not legally binding but provide scientifically grounded thresholds for assessing contamination risks [197].

The FDA regulates most PBMAs as “food” under the Federal Food, Drug, and Cosmetic Act (FFDCA). The U.S. Department of Agriculture (USDA) oversees meat alternatives that contain egg or dairy ingredients or are marketed as direct meat product substitutes [198]. The FDA’s Bacteriological Analytical Manual (BAM) contains general testing methods for foodborne pathogens, but there are no PBMA-specific limits (https://www.fda.gov/food/laboratory-methods-food/bacteriological-analytical-manual-bam accessed on 5 November 2026) [199].

USDA (Food Safety and Inspection Service)-FSIS guidelines for ready-to-eat foods may apply to cooked PBMAs but they are not tailored to plant-based products (https://www.fsis.usda.gov/ accessed on 5 November 2026) [200].

## 6. Current Availability, Market Situation, and Market Development of Protective Cultures for Plant-Based and Cooked Meat Products

The growth drivers for plant-based products are diverse. The ongoing expansion of the market for protein alternatives (which has a compound annual growth rate (CAGR) of 3.68% globally) is a key factor that deserves attention. As a result, the product range is expanding, but plant-based protein alternatives are also highly perishable, necessitating research into methods to improve their shelf life. Furthermore, the adoption of protective cultures is expected to be driven by demand for improved food safety, reduced perishability, and longer shelf life (Choudhury 2025, https://www.futuremarketinsights.com/reports/food-and-beverage-protective-cultures-market accessed on 5 November 2026) [201]. A number of protective cultures for commercial use in PBMAs have been developed in recent years (Table 3). Suppliers offer both new product lines and conventional starter cultures for the production of sausage substitutes. In the plant-based milk product sector, entire product lines of starter cultures have been available for some time (IFF 2024, https://bioscience.iff.com/solutions/food-and-beverages/plant-based-alternatives accessed on 5 November 2026; Novonesis, https://www.novonesis.com/en/biosolutions/food-and-beverages/plant-based-foods/plant-based-meats accessed on 5 November 2026) [202,203].

The protective cultures available on the market for PBMA products are comparable to the LAB used in products of animal origin. The exact strains of the protective culture products available on the market are often not specified, and in published studies, they are designated by internal isolate numbers or exclusively by species names, without providing unique identification numbers for strain classification.

Furthermore, the existing literature on protective cultures primarily focuses on demonstrating their effectiveness in conventional, animal-based products. For PBMA applications, the effectiveness of commercially available products is currently based solely on information provided by the respective manufacturers, as no independent, published studies confirming their efficacy are available.

Examples of such organisms include *S. xylosus DD-34* and *Lt. sakei* (B-2 Safe Pro^®^). While LAB are already well-established in PBMA, probiotics and bacteriocin-producing *Bacillus* spp. are emerging as potential candidates, although their specificity and efficacy in plant-based matrices still need to be further optimized (Novonesis, https://www.novonesis.com/en/biosolutions/food-and-beverages/plant-based-foods/plant-based-meats, https://www.meatcracks.de/media/m-cultures_meat_de_ex.pdf accessed on 5 November 2026) [18,25,53,202,207].

The use of protective cultures in PBMA products is intended to inhibit the growth of foodborne pathogens and extend shelf life by controlling spoilage microorganisms. In addition, a growing variety of culinary traditions are being utilized to improve the sensory properties of foods, particularly their texture and aroma. Furthermore, it has been demonstrated that LAB exert a probiotic effect, thereby improving the nutritional value of foods, regulating serum cholesterol levels, preventing intestinal infections, strengthening the immune system, and alleviating antibiotic-associated diarrhea [208,209,210].

It is important to recognize that the development of new food cultures depends on meeting numerous conditions and requirements, thereby transforming a purely scientific approach into a marketable product. First, it is essential to ensure comprehensive health and safety measures, such as those required by GRAS or QPS status. Furthermore, it is essential to ensure that no metabolic activity occurs at low temperatures, as this could potentially lead to undesirable sensory effects for the consumer. At the same time, a sufficient quantity of potentially metabolically active bacteria must be maintained throughout the entire duration of cold storage, even in the event of short-term interruptions in the cold chain. Protective cultures must inhibit the growth of toxic-infectious bacteria within a temperature range of at least 7–8 °C and should ideally suppress a broad spectrum of such target organisms. Furthermore, potential food cultures should be characterized with whole-genome sequencing (WGS) and tested for antibiotic resistance as well as biogenic amines [211,212].

Current developments point toward a shift away from the general use of food cultures toward tailored, matrix-specific development strategies that also reflect the type of enhanced protective effect of LAB cultures that has been observed under vacuum conditions, whereas this effect appears to be less pronounced in modified-atmosphere packaging [212,213].

The global market for protective cultures for heat-treated foods is currently limited, but innovations are expected to expand these markets. The main barriers to growth are regulatory and labeling requirements in certain countries (Choudhury, https://www.futuremarketinsights.com/reports/food-and-beverage-protective-cultures-market accessed on 5 November 2026) [201,214].

## 7. Data Gaps and Conclusions

Although most of the scientific literature to date has focused on the control of *B. thermosphacta* and *Listeria monocytogenes*, protective cultures have established themselves as an effective, clean alternative for controlling spoilage and pathogenic microorganisms in a wide variety of products of animal origin. However, this review reveals that key areas of application remain under-researched. Currently, no specific studies have been conducted on PBMAs. Commercially available protective cultures are primarily designed for conventional meat products and raw sausages, and their effectiveness in plant-based matrices has not been adequately demonstrated. This is particularly relevant because plant-based protein matrices have specific associated spoilage microorganisms, primarily spore-forming bacteria such as *Bacillus* and *Clostridium* species. The population dynamics of these bacteria differ from those of traditional meat products. These bacteria are also largely tolerant of acid-based inhibitory mechanisms due to their spores. In addition, knowledge gaps remain regarding the characteristics of relevant protein sources (e.g., soy, peas, and mycoprotein) at a strain-specific level, as well as challenges in transferring laboratory-based findings to an industrial scale. Furthermore, protective cultures have primarily been considered single strains in research thus far. Systematic investigations into multi-strain combinations or coupling cultures with bacteriocins are rare, despite existing studies indicating the synergistic effects of such combinations. The same applies to process engineering approaches, such as the encapsulation of protective cultures, which would enable their use prior to heating. Whole-genome sequencing (WGS) might enable the identification of genes encoding desirable functional traits in candidate protective cultures. With further research, the corresponding gene products could be overexpressed in the producing strain or delivered via established encapsulation systems, while bacterial extracellular vesicles represent a more distant, still experimental delivery option.

Future research should systematically examine the functional overlaps between protective cultures, probiotics, synbiotics, and postbiotics. Of particular interest are the metabolic effects of short-chain fatty acids (SCFAs), the immunomodulatory properties of which are increasingly well documented. They are involved in modulating inflammatory processes relevant to infectious and non-communicable diseases. It remains to be clarified whether the concentrations of metabolites actually produced by protective cultures in fermented foods exert physiologically relevant immunological effects, but this represents a promising interdisciplinary research direction. Given the rapid growth of the plant-based alternatives sector, an increase is expected regarding the demand for customized protective cultures. Therefore, filling the knowledge gaps outlined above is a prerequisite for establishing scientifically sound safety and quality standards for the next generation of plant-based and cooked meat products.

## Figures and Tables

**Table 2:** Bacteriocin-producing microorganisms examined as protective or starter cultures in meat products. Sources: [41,42,43,44,45,46,47,48,51,52,74,75,76,77,78,79,80,81,82,83,84,85,86,87,88].

Bacteriocin	Bacterial Strain	Source
bacteriocin gen papA	*P.acidilactici* LMQS 154.1; *Lc. lactis* DSM 20729; *P. pentosaceus* LMQS 331.3	[52]
bavaricin MN	*Lt. bavaricus* MN; *Lt. sakei* MN	[74]
brevicin 27	*Lv. brevis* SB27	[75]
carnobacteriocin A	*C. piscicola* LV17A	[75]
carnobacteriocin B1	*C. piscicola* KLV17B	[75]
carnobacteriocin B2	*C. piscicola* KLV17B; LV17B	[75]
curvaticin FS-47	*Lt. curvatus* FS47; LTH1174	[75]
curvaticin L442	*Lt. curvatus* L442	[47]
divergicin 750	*C. divergens* 750	[75]
divergicin A	*C. divergens* LV13	[75]
enterocin 416Kl	*Ec. casseliflavus* lM416K1S	[75]
enterocin A- and B	*Ec. faecium* CTC492	[43]
enterocin A, pediocin L50A and L50B	*Ec. faecium* PCD71	[77]
enterocin AS-48	*Ec. faecalis* A-48-32	[78]
enterocine	*Ec. faecium* SE920	[79]
lactocin 705 and AL705	*Lt. curvatus* CRL705	[76]
lactocin S	*Lt. sakei* 148; L45; V18	[44,75]
leucocin A	*Leuc. carnosum* TA11a; DMRICC 4010; *Leuc. gelidum* UAL187; *Leuc. mesenteroides* L124; TA33a	[47,75]
leucocin B	*Leuc. carnosum* DMRICC 4010	[41]
leucocin C	*Leuc. carnosum* 4010	[45]
nisin	*P. acidilactici* LMQS 154.1 *Lc. lactis* BB24: subsp. *lactis* I23; subsp. *hordinae* E91; DSM 20729;	[52,75,80]
nisin Z	*Lc. lactis* WNC20	[75]
pediocin	*P. acidilactici* NCIMB 700993; P02 (JBL1097); PA-2; (B-LC-20); *Lt. bavaricus* MI-401; *S. xylosus* DD-34	[81,82,83,84]
pediocin AcH	*P. acidilactici* H (JBL1095); PAC 1.0	[51]
pediocin l.50	*P. acidilactici* l.50	[75]
pediocin PA-1	*P. acidilactici* HA-6111-2; MCH14; PAC 1.0; *P. pentosaceous* Z102	[75,85,86]
piscicolin 126	*C. piscicola* JG126	[75]
piscicolin v1a	*C. piscicola* V1	[75]
plantaricin A	*Lp. plantarum* CTC305	[75]
sakacin A	*Lt. sakei* LB 706	[75]
sakacin C2	*Lt. sakei* C2	[49]
sakacin G	*Lt. sakei* L2512	[42]
sakacin K	*Lt. sakei* CTC 494; LTH673; 674	[88]
sakacin P	*Lt. sakei* 1151; LTH673; 674	[75]
sakacin Q	*Lt. curvatus* ACU-1	[46]
sakacin T	*Lt. curvatus* 2711	[42]
sakacin X	*Lt. curvatus* 2711	[42]
sak X, sak Tα, sak Tβ, sak P	*Lt. curvatus* 54M16	[87]

**Table 3:** Commercially available protective cultures for plant-based meat alternatives and cooked meat products. For plant-based meat alternatives, the reported efficacy is based solely on manufacturer specifications. Currently, there are no published, independent studies evaluating these cultures in their intended applications.

PC	Application	Product	Effect
*Lt. sakei*	plant-based meat alternatives	Vertera^®^ SafePro^®^ 01	✓ keep products fresher for longer ✓ a safer fermentation process ✓ reduce reliance on additives
*Leuc. carnosum*	plant-based meat alternatives	Vertera^®^ SafePro^®^ 02	✓ keep products fresher for longer
			✓ enhanced product stability ✓ reduce reliance on additives
*Lc. lactis*	plant-based meat alternatives	Vertera^®^ SafePro^®^ 03	✓ protect against spoilage ✓ extend freshness
			✓ microbial stability
*Leuc. carnosum*	fresh vegan meat substitute	M-CULTURE^®^ Safe Vegan	✓ displacement effect
	products based on peas		
*Lp. plantarum*	fermented plant-based products	M-CULTURE^®^ plant LEG MA	✓ mild to strong acid formation
*Propionibacterium freudenreichii* subsp. *shermanii*, *Ls. rhamnosus*	vegan products in general	Holdbac YM-Vege	✓ inhibit mold growth in fermented plant-based products
*Lt. sakei*	fermented, sliced cooked ham and cooked sausages	Bactoferm^®^ Safepro^®^ B-2	✓ reduce the growth of spoilage and pathogenic bacteria such as native lactic acid bacteria and *L. monocytogenes*
*Lt. curvatus* DSM 18775	cooked and sliced meat products and Wiener sausages	SafePro^®^ B-LC-48	✓ inhibit the growth of *L. monocytogenes*
*Lt. sakei*	cooked or cured meat products	B-2 SafePro^®^	✓ improve food safety and/or potentially extend shelf life
*Lt. sakei*, *S. xylosus*	fresh meat, cooked and sliced products	Lyocarni BXH-69	✓ inhibition of spoilage bacteria
*C. divergens*, *C. maltaromaticum*, *Lt. sakei*	fresh meat, cooked and sliced products	Lyocarni BOX-74	✓ inhibit the growth of *L. monocytogenes*
*Carnobacterium* spp., *Lt. sakei*	fresh meat, cooked and sliced products	Lyocarni BMX-37	✓ inhibit the growth of *L. monocytogenes*
*Carnobacterium* spp., *Lt. sakei*	fresh meat, cooked and sliced products	Lyocarni BOM-13	✓ inhibit the growth of *L. monocytogenes*
*Carnobacterium* spp., *Lt. sakei*	fresh meat, cooked and sliced products	Lyocarni BXH-69	✓ inhibit the growth of *L. monocytogenes*
*Carnobacterium* spp.	fresh meat, cooked and sliced products	Lyoflora FP-18,	✓ inhibit the growth of *L. monocytogenes*
*Carnobacterium* spp.	fresh meat, cooked and sliced products	Lyoflora FP-50	✓ inhibit the growth of *L. monocytogenes*

Sources: Novonesis, https://www.novonesis.com/en/biosolutions/food-and-beverages/plant-based-foods/plant-based-meats, M-Food, https://www.meatcracks.de/media/m-cultures_meat_de_ex.pdf, U.S. Food & Drug Administration, https://www.fda.gov/files/food/published/GRAS-Notice-GRN-760-Lactobacillus-curvatus-DSM-18775%20.pdf, Netropolitan, https://netropolitan.co.nz/wp-content/uploads/2018/11/PI_B-2_501116_EN.pdf, IFFA, https://iffa.messefrankfurt.com/frankfurt/en/exhibitor-search.detail.html/sacco-srl/mf_1_0012553884_4396982_10000012202501.html, accessed on 5 November 2026 [8,25,28,202,204,205,206].

## Data Availability

No new data were created or analyzed in this study. Data sharing is not applicable to this article.

## Supplementary Materials

The following supporting information can be downloaded at https://www.mdpi.com/article/10.3390/foods15213984/s1: Figure S1: This figure illustrates the initial process of the literature search for this review. Created in BioRender. Hennes, T. (2026). https://BioRender.com/etnz2ud accessed on 5 November 2026. Table S1: The table shows an overview of all starter and protective cultures approved according to QPS/EFSA status for meat and vegetarian/vegan products. Table S2: This table provides an exemplary overview of commercially available plant-based meat products, detailing the employed protein sources and representative brand examples.
