# Supplementary material for: Protective Cultures in Meat Products and Plant-Based Meat Alternatives: Prospects and Challenges
Source: Foods. 2026 Nov 6;15(21):3984. doi: 10.3390/foods15213984 (PMC13762629; doi:10.3390/foods15213984)
Supplement: Supplementary file 1 [file foods-15-03984-s001.zip › foods-4495552-supplementary.pdf]

Supplemental Figure S1: This figure illustrates the initial process of the literature search for this review. Created in BioRender. Hennes, T. (2026) https://BioRender.com/etnz2ud). https://biorender.com/mba49jy.

Supplement Table S1

The table shows an overview of all starter and protective cultures approved according to QPS/EFSA status for meat and vegetarian/vegan products. Abbreviation: EFSA: European Food Safety Authority; QPS: Qualified Presumption of Safety. * VO (EU) 2025/138, **and QPS [27, 29], *** GRAS [66]

Supplement Table S2 provides an exemplary overview of commercially available plant-based meat products, detailing the employed protein sources and representative brand examples.

Supplement Figure S1

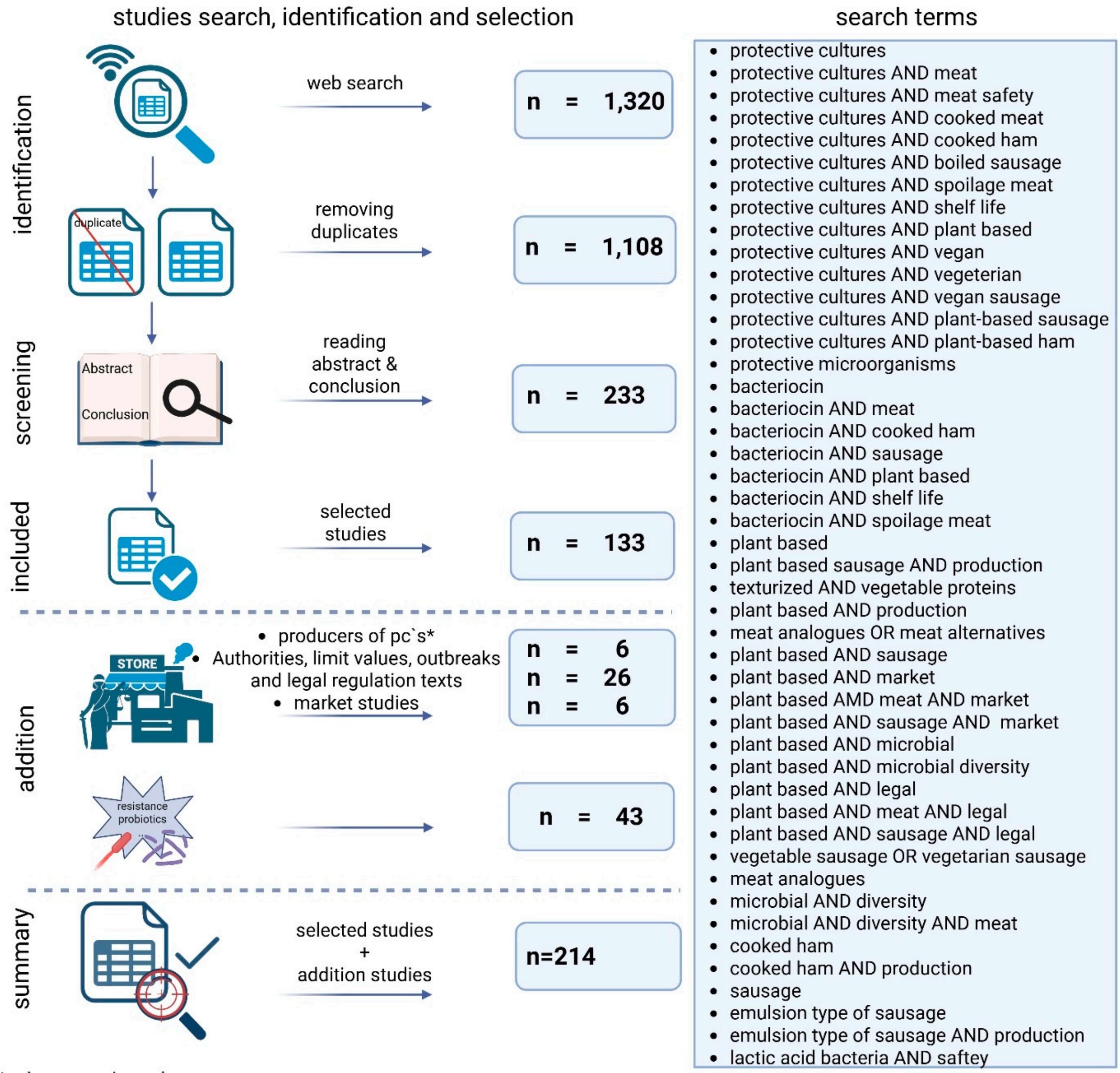

*pc`s: protective cultures

Supplement Table S1

Microorganism	Protective	Starter	Meat	Vegan	Strain	Application	Function	*Listeria* inhibition	Pathogen inhibition
*Lacticaseibacillus casei**	x			x	LC-01, DN-114 001	meat alternatives, vegan spreads			
*Carnobacterium maltaromaticum***	x		x		CB1, LF1	vacuum meat/ fish products			
*Leuconostoc mesenteroides***	x			x	Lm1, DSM 20241	sauerkraut, vegan ferments			
*Lactiplantibacillus plantarum***		x		x	299v, CCM 7039	yogurt, silages, vegan ferments			
*Lactococcus lactis***		x		x	subsp. *cremoris* MG1363, NCDO 712	cream cheese, vegan alternatives			
*Pediococcus acidilactici***		x	x	x	PA-1, ATCC 8042	raw sausage, fermented soy products			
*Propionibacterium freudenreichii***		x		x	PfS1, DSM 4902	Emmental, vegan cheese			
*Latilactobacillus sakei***	x	x	x		LS-25, DSM 15831	raw sausage (salami, pepperoni)	rapid lactic acid formation, color stabilization	++	*L. monocytogenes, E.coli*
*Lactobacillus curvatus***	x		x		Lc-11, ATCC 51436	raw sausage, fermented meat products	bacteriocin production (sakacin), mold inhibition	+++	*L. monocytogenes, Clostridien*
*Staphylococcus xylosus****	x	x	x		Sx-15, DSM 20266	raw sausage (surface culture)	nitrate reduction, aroma formation, color formation		*Staphylococcus aureus*
Staphylococcus carnosus	x	x	x		Sc-7, DSM 20501	cooked sausage, cooked ham	nitrate reduction, reddening		*Clostridien*
*Pediococcus pentosaceus***	x	x	x	x	PP-1, ATCC 43200	raw sausage, fermented soy products	rapid lactic acid formation, stabilization	+	*E. coli, Salmonellen*
*Limosilactobacillus fermentum***	x		x		LF-3, DSM 20052	raw sausage, dry sausage	antioxidant effect, lipid stabilization	++	*L. monocytogenes*
*Lacticaseibacillus paracasei***	x		x		Lpc-4, DSM 5622	cooked sausage, meat spreads	immunomodulation, pathogen inhibition	+++	*L. monocytogenes, Staphylococcus*
*Latilactobacillus sakei***	x		x		LaS-1, DSM 15831	raw sausage, fermented meat products	sakacin production, mold inhibition	+++	*L. monocytogenes*
*Carnobacterium divergens***	x		x		CD-1, DSM 20633	vacuum meat products	bacteriocin production (anti-*Listeria*)	+++	*L. monocytogenes*
*Lactiplantibacillus plantarum***	x	x	x	x	Lp-115, DSM 12028	raw sausage, fermented vegetable products	biofilm inhibition, pH stabilization	+++	*L. monocytogenes, E.coli*

Supplement Table S2

Product Type	Common Protein Sources	Example Brands/Products
Mortadella	sunflower, pea, mung bean	Rügenwälder Mühle, Billie Green, VegaVita
Frankfurter	wheat gluten, pea, soy, sunflower	Green Force, Bon Vegan, die Ohne
Leberkäs	wheat gluten, pea, sunflower	Green Force, GAUDI GUSTL
Cooked ham	wheat gluten, soy, pea, sunflower	LaVie, Green Force, Accro
Salami	wheat gluten, soy	Rügenwälder Mühle, Billie Green, Planeo, Renegade Foods
Raw ham	wheat gluten	Billie Green
Bacon/Bacon bites	wheat gluten, pea, soy, sunflower	Rügenwälder Mühle, LaVie, Billie Green
Grilled sausage	soy, pea, rice	Garden Gourmet, Beyoned Meat, Redfine meat
White pudding	pea, kidney beans, soy	Rügenwälder Mühle, granoVita
Minced Meat	pea, soy, sunflower, mycoprotein	Garden Gourmet, Redfine Meat, Impossible Foods, Quorn
Burgers/Patties	pea, rice, soy, wheat gluten, corn	Beyond Meat, The Vegetarian Butcher, Garden Gourmet
Schnitzel	pea, soy, wheat gluten, corn, rice, sunflower	Veganz, The Vegetarian Butcher, Vegini
Steak	soy, wheaten gluten	Planted, Juicy Marbles, Redfine Meat, Chunk Foods
Chicken breast fillet	wheaten gluten, soy, pea	Green Mountain, Vivera, Accro
Pulled Chunks	soy, rice, pea, chick-pea	Planted, Vegini, Green Mountain
Gyros/Kebab	soy, wheat gluten, pea, broad bean	Tofutown, Planted, Davert, edgy veggie
Chicke' n nuggets	soy, pea, corn, wheat gluten, broad bean	Like Meat, Green Cuisine, Beyond meat, Impossible Foods
Grill skewers	wheat gluten,broad bean, pea	GreenLegend, Planted

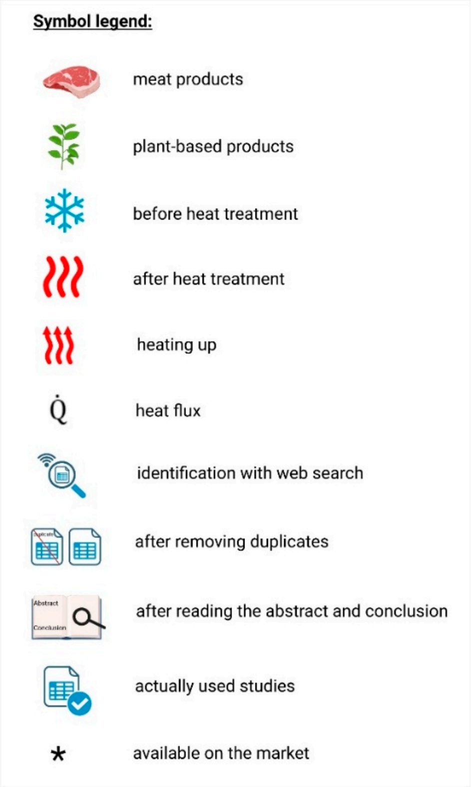

Created in BioRender. Hennes, T. (2026) https://BioRender.com/3jh8tav
